# The Effect of Cage Space on Behavior and Reproduction in Crl:CD1(Icr) and C57BL/6NCrl Laboratory Mice

**DOI:** 10.1371/journal.pone.0127875

**Published:** 2015-05-28

**Authors:** Brianna N. Gaskill, Kathleen R. Pritchett-Corning

**Affiliations:** 1 Purdue University Department of Comparative Pathobiology, West Lafayette, Indiana, United States of America; 2 Harvard University Faculty of Arts and Sciences, Office of Animal Resources, Cambridge, Massachusetts, United States of America; 3 Charles River, Wilmington, Massachusetts, United States of America; Université Pierre et Marie Curie, FRANCE

## Abstract

Recommendations for the amount of cage space required for female mice with litters were first made in the 2011 *Guide for the Care and Use of Laboratory Animals*. We hypothesized that if a difference in mouse behavior and reproduction exists within the limits of commercially available caging, this difference would be detected between the smallest and largest cages. C57BL/6NCrl and Crl:CD1(Icr) breeding mice were randomly assigned to a cage treatment: LP 18790 (226cm^2^); A RC1 (305cm^2^); A N10 (432cm^2^); T 1291 (800cm^2^) and a breeding configuration: single (male removed after birth); pair (1 male + 1 female); or trio (1 male + 2 females) in a factorial design for 12 weeks. All cages received 8-10g of nesting material and nests were scored weekly. Pups were weaned between post-natal day 18 and 26 and were weighed at weaning. Adult behavior and location in the cage were recorded by scan samples every 30 min over 48 hr of video recorded on PND 0-8 and PND 14-21 when pups were in the cage. Press posture and play behavior were recorded by 1/0 sampling method. Cage space did not significantly alter typical reproductive measures. Pups in the smallest cage played less than in the other cages. Adults in the smallest cage displayed more press posture than in the two largest cages. Mice in the largest cage spent more time under the feeder than in other areas of the cage. Nest score was also the highest in the largest cage. Housing breeding groups of mice in a range of commercially available cage sizes does not affect reproduction but behavioral measures suggest that the smallest cage tested, LP 18790, may be stressful for outbred mice when pups are present.

## Introduction

The *Guide for the Care and Use of Laboratory Animals*, usually abbreviated as the *Guide*, has defined the standard of care of laboratory animals in the United States since its initial publication in 1963. The *Guide* has been revised 7 times since1963, including in 1965, 1968, 1972, 1978, 1985, 1996, and 2011 as new information or changes in best practice made revision necessary. The two latest iterations of the *Guide*, the 1996 *Guide* and the 2011 *Guide* contain more information about mice than previous editions, reflecting their increasingly vital role in biomedical research. The 1996 *Guide* and 2011 *Guide* provide cage space recommendations for mice virtually unchanged from the 1972 *Guide* (Table 1) but no edition of the *Guide* before 2011 has ever directly addressed the space requirements for females with litters [1]. Faced with this omission, but concerned about welfare given the explosion of genetically modified mice that occurred beginning in the mid-1980s [2], institutions created their own guidelines for breeding rodents based on both the space requirements in the 1996 *Guide* and their professional judgment. These space recommendations gradually became a working standard within the biomedical research community. Historically, a breeding trio (1 male and 2 females) of mice plus a litter from each female was considered the equivalent of 5 mice in a cage, the maximum number allowed in a typically-sized plastic mouse cage (426–436 cm^2^). This widely-adopted working standard allowed institutions to breed 2 females with 1 male in standard cages, a practice thought to ensure pup survival and increase overall production, especially with genetically modified mice or inbred strains with small litters.

**Table 1:** Evolution of cage space requirements for mice through versions of the *Guide*.

Version of the *Guide*	Number/weight of mice	Housing area per animal
1963	1–6/20 g	92.9–650.3 cm^2^/animal
	10–20/20 g	46.5–92.9 cm^2^/animal
1972 and 1974	<10 g	39 cm^2^
	10–15 g	52 cm^2^
	16–25 g	77 cm^2^
	>25 g	97 cm^2^
1978 and 1980	<10 g	39 cm^2^
	10–15 g	52 cm^2^
	16–25 g	77 cm^2^
	>25 g	97 cm^2^
1985 and 1996	<10 g	38.71 cm^2^
	10–15 g	51.62 cm^2^
	16–25 g	77.42 cm^2^
	>25 g	96.78 cm^2^
2011	<10 g	38.7 cm^2^
	10–15 g	51.6 cm^2^
	16–25 g	77.4 cm^2^
	>25 g	≥96.78 cm^2^
	Mother and litter	330 cm^2^

The latest revision of the *Guide* was published in 2011 and directly addressed cage size for females with litters [3]. Females with litters were assigned their own recommended space of 330 cm^2^. This space recommendation appears to have been taken directly from European regulations, since the recommendation is identical, but in those regulations, this space requirement applies to both breeding pairs and trios of inbred mice [4]. Neither the *Guide* nor the European regulations have cited literature to support the space requirements suggested for females with litters. Changes made to the *Guide* were likely intended to improve the welfare of breeding rodents based on the perception that increased density causes crowding stress in mice, but few studies have been conducted to directly investigate the cage space needs of breeding mice [5–7].

The extreme behavioral and genetic plasticity of mice makes it difficult to readily define the minimum or maximum amount of space required for a mouse. Although not generally considered a peripatetic species, mice readily cover kilometers daily as part of regular patrols of their home range [8]. Mice in grain fields in Australia can have territories of as little as 2 m^2^ or up to 8 hectares [9], but mouse plagues have been described where the estimated population was more than 70,000 individuals per acre [10]. Mice with ample resources, such as those who live in human homes, seldom venture more than a few meters from the nest [11]. All of these described territories are significantly larger than the spaces provided to laboratory mice. For example, in the plague conditions described above each animal still had a potential territory of approximately 85 in^2^ or 548 cm^2^.[12–14]

The use of space by laboratory mice has been a subject of investigation for almost the moment scientists acquired mice from mouse fanciers for their laboratories. Cage sizes and materials have changed through the years, but how many mice may be kept in a “standard” cage has been investigated using many different means. The primary driver of these investigations has been observation and interpretation of mouse housing conditions by people caring for the mice. The study of this question involves two separate factors, one being the number of mice in the cage (group size) and the second being the amount of physical floor space available to the mice (cm^2^/animal). Mice are social animals, where males and females defend established territories [15], so group size is an important influencer of mouse behavior [16]. Much of the peer-reviewed cage space literature confounds space per mouse with group size, primarily by using only one size of cage and then adding or subtracting animals to reach the desired floor space per mouse (see review by [17]). Investigators have also created custom caging to study cage space, and provided different forms of enrichment and structural complexity often not reported in detail, thus making duplication of their work difficult [18–26]. The variability in caging and enrichment makes broad application of the conclusions reached by much of the published cage space research challenging.

When research has been performed using commercially available caging and controlling for both group size and space per mouse, it has typically been performed with single sex groups, not breeding animals, as the interest of investigators is in behavioral problems such as fighting or in altered physiological parameters that would make the mice unsalable or unusable for experiments [5,6,27–32]. When groups of same-sex mice are queried as to their preferences, they do seem to prefer access to more space [12–14]. Their use of that space reveals their true purpose, however, which is not leaving, but going. To clarify, mice do not stay in the extra space they worked to access, instead, they satisfy their curiosity about the new space and add it to their patrol routes. They spend most of their time in a smaller area, not necessarily the new area they worked to access. When considering space offered by larger cages, this may mean that mice will patrol the perimeter of a larger cage, but spend most of their time near the nest.

For commercial breeders, the primary interest is in production of offspring by breeding animals, as they comprise the majority of cages held. In research facilities, fewer animals are used as breeders, but most researchers do breed mice to some extent. Breeding mice are less commonly addressed in the cage space literature, despite their abundance. In some early work, large groups of mice were confined to custom-built cages and behavior, fighting, and reproductive output were measured in these semi-natural situations and data were collected from wild populations as well [10,15,33–35]. Harvey and Chevins crowded pregnant mice in mid-pregnancy by housing them in high density situations with groups of males [36], which is not a typical breeding configuration. Krackow used groups of pregnant females without males as the crowded condition so animals only littered once, which is, again, not typical for breeding mice [37]. More recently, Whitaker *et al*. examined breeding performance of trios of C57BL/6NTac mice kept in one of two cage sizes [6,7]. In neither study were reproductive effects noted at a statistically significant level, although some inconsistent changes in offspring response in behavioral assays were noted [6,7]. More effects were seen when cage size was also tested with enrichment in a factorial design, but cage size was less of a factor than the presence or absence of enrichment [7]. In fact, the most consistent result found in studies testing different amounts of cage space for either breeding or stock animals, is that mice or pups in larger cages tend to weigh less (see review by [17]. This is more likely an effect of cold stress in larger cages with less total mouse body mass and little or no nesting material, than an actual effect of floor space [38].

In the present study, the behavior and reproduction of breeding mice housed in a range of 4 differently-sized commercially available cages were examined. We hypothesized that if a difference in behavior and reproduction exists within the limits of commercially available caging, this difference would be detected between the smallest and largest cages. To test this hypothesis, we looked at various measures of reproduction and behavior using two commonly available mice, the small and less productive inbred C57BL/6NCrl (B6), and the large and extremely fertile outbred Crl:CD1(Icr) (CD1).

## Materials and Methods

**Fig 1:**
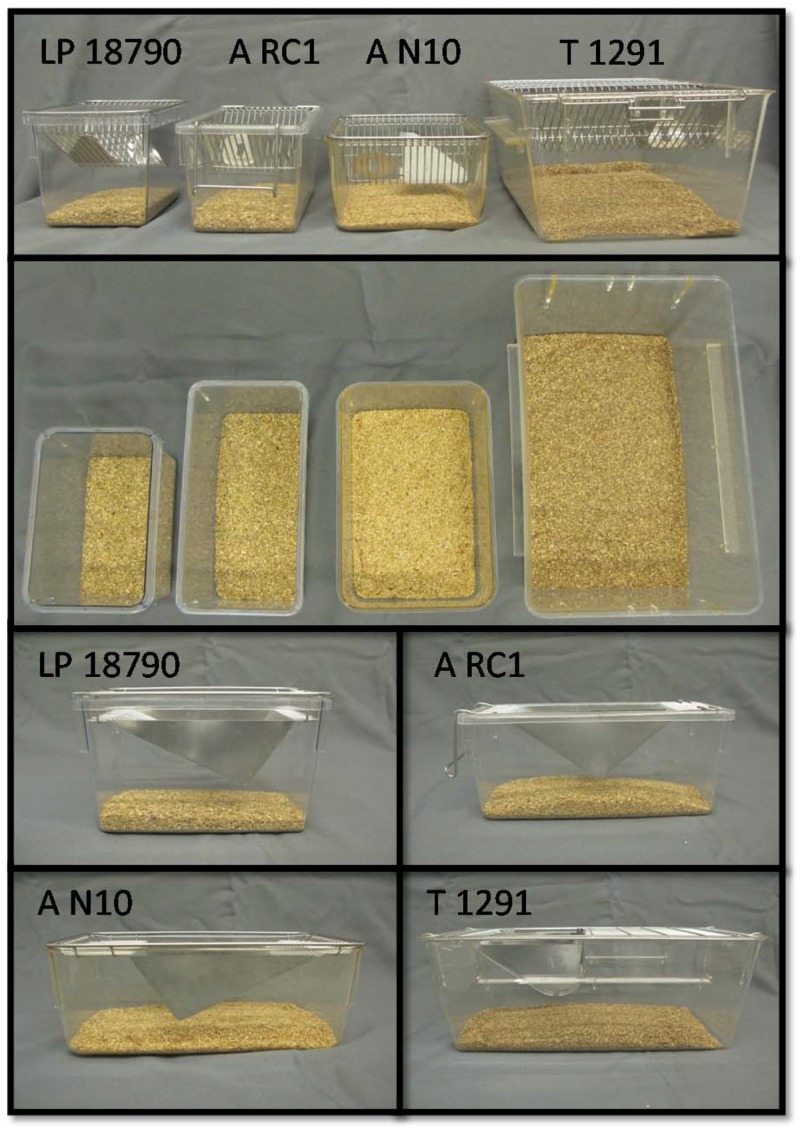
Images of the four different cages used to test if the size of cage altered reproduction or behavior in breeding mice. Top: view of the front of the cages. Cage size increases from left to right. Middle: top view of cages; Bottom grid: Side view of cages used.

All work was conducted at Charles River’s AAALAC-accredited Wilmington, MA, (Location 1) and Raleigh, NC, (Location 2) facilities and was approved by Charles River’s Institutional Animal Care and Use Committee (P01112012). Animals were free of a list of common mouse infectious agents at the start of the study; further details may be found at http://www.criver.com/files/pdfs/rms/hmsummary.aspx. No further health monitoring was performed on animals on study. Females were approximately 49 days of age (± 3d) at study start, while males were approximately 56 days of age (± 3d). Mice used in Location 2 were sourced from that location, while mice used in Location 1 of the study were sourced from Charles River’s Kingston, NY, facility. Mice were housed in solid-bottomed cages of varying sizes (Table 2, Fig 1). Cages were sourced from Tecniplast (T 1291; 42.4 x 26.7 x 18.3 cm; floor area of 800 cm^2^; West Chester, PA), Ancare (A N10; 29.2 x 19.0 x 12.7 cm; floor area of 432cm^2^; and A RC1; 26.5 x 11.4 x 14.8 cm; floor area of 305cm^2^; Bellmore, NY), or Lab Products (LP 18790; 23.5 x 15.2 x 15.6 cm; floor area of 226 cm^2^; Seaford, DE). Three of the four cage sizes used can be purchased directly from the manufacturer; one Ancare cage is produced solely for Charles River (A RC1). Cages were placed on rolling racks (Metro, Wilkes-Barre, PA) for the duration of the experiment.

**Table 2:** Differences between cage area provided in this study and the amount of space recommended by the 2011 *Guide*.

		Female and litter 2011 *Guide*	Breeding pair 2011 *Guide*	Breeding trio 2011 *Guide*
	Cage area (cm^2^)	330	427	757
LP 18790	226	-104	-201	-531
A RC1	305	-25	-122	-452
A N10	432	102	5	-325
T 1291	800	470	373	43

All cages were open to the environment (no individually ventilated caging was used). All cages were bedded with heat-treated chipped hardwood bedding (Beta Chip; NEPCO, Warrensburg, NY), and mice were also provided with 8–10 g of a long-fiber paper nesting material (EnviroDri, Shepherd Specialty Papers, Watertown, TN) at the weekly cage change. Food (5L79, LabDiet, St. Louis, MO) and ultrafiltered hyperchlorinated water were provided *ad libitum* via water bottle. The light cycle was 12:12 light:dark (on at 0630, off at 1830), humidity was maintained between 30–70% and temperature ranged between 19–22°C. Cages were checked daily to monitor animal health; ill animals were removed from study but the cage was only replaced at Location 1. In both facilities, rats and mice on cage density studies were housed in dedicated areas that did not contain other, non-study animals. Since animals on study were of similar microbial status, they were concurrently housed in the same study rooms, but on different racks, based on previous work showing co-housing has no effects on reproduction in either species [39]. Any ill animals and all animals at the end of the study were euthanized with CO_2_, inhaled to effect.

**Fig 2:**
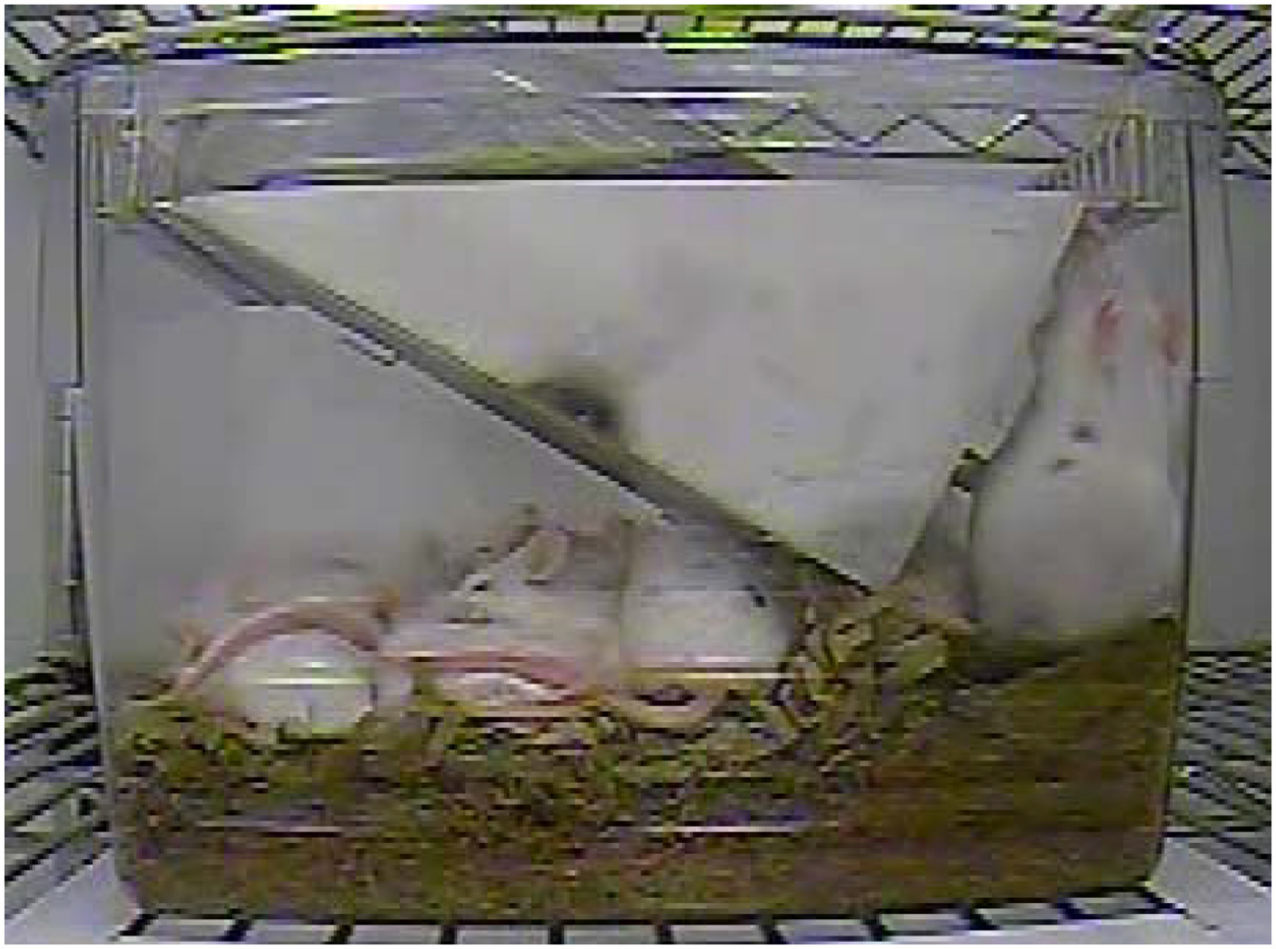
Image illustrating “press posture”. This behavior is characterized by the placement of the ventral surface of the body in the corner of the cage and a subsequent lack of movement. Mice were often observed to fall asleep in this position.

The experiment was split into 2 arms. Location 1 focused on behavior in 48 reproductively successful cages of breeding mice (112 mice total at the start of the experiment, divided evenly between B6 and CD1). Animals were allocated to treatment groups using the random integer set generator function found at random.org. Cages were allocated to breeding treatment as follows: 16 cages (8 CD1 and 8 B6) as trio matings with 1 male and 2 females; 16 cages (8 CD1 and 8 B6) as pair matings with 1 male and 1 female; and finally, 16 cages (8 CD1and 8 B6) in which the male was removed 24–48 h after a litter was born, to model a situation in which a female rears pups by herself. For solo females, the male was reintroduced after the pups were weaned only if the female had not become pregnant at the postpartum estrus. Location 1 animals were filmed for two 24 hour periods, once during PND 0–8 of pups and once during PND 14–21 of pups. The video was evaluated by one investigator (BNG), who could not be blinded to treatment due to the nature of the study. Adult behavior was instantaneously scan sampled every 30 minutes in both the PND 0–8 and 14–21 videos. Tables 3 and 4 contain the detailed ethogram used. 1/0 sampling (yes/no), during a 2 minute sampling interval every 30 minutes, was utilized for adult press posture and pup play behavior. Pup play behavior however was not scored during the PND 0–8 video since neonates are not mobile enough for recognized play. Adult press posture (Fig 2) was scored in both videos. Reproductive data collected included: litter size at birth, litter size at weaning, weaning weight, and whether females were pregnant at the end of the study. From these data, we calculated the interlitter interval of litters 1 and 2, pup mortality, and the number of pups weaned/female/week (production index; PI). Nest scores were also collected weekly based on a 1–5 scale [40]. Briefly, a score of 1: was manipulated material but no central nest site was evident; 2: was a flat nest; 3: was a cup nest; 4: was an incomplete dome; 5: was a complete and enclosed dome.

**Table 3:** General behavioral budget ethogram.

General Activity	General locomotion	All locomotive behavior performed on the cage lid, climbing up the cage bars by the food hopper to reach the lid, and locomotion on the floor of the cage.
	Rearing	Animals on the floor of the cage would rear up on their hind legs. Rearing was usually accompanied by sniffing movements.
	Sniffing	Sniffing was also performed against the cage floor (ground), or in between the bars of the cage lid.
Inactive	Inactive-in-nest	The animal within the nest, due to camera angles, cannot clearly be seen but no movement within the nest can be detected. It is assumed that the animal is sleeping within the nest. This is distinguishable from other behaviors within the nest because movement within the nest or of the nest itself is not observed
	Still and alert	The animal was sitting curled up, but in contrast to sleep, the face was lifted. The animal either sat motionless, or would appear to be orientating its head to sounds outside of the cage.
	Sleep	The animal was motionless, and either lying curled up on its side, or sitting curled up, with its face tucked into its body and out of sight of the camera. Occasionally interrupted by brief single twitches.
Maintenance	Eating	The animal would rear up to gnaw at food pellets through the bars of the hopper. The forepaws would usually be used to hold the food pellet steady.
	Drinking	The animal would rear up and lick the nipple drinker
	Grooming self or other adult	All grooming behavior including licking the fur, grooming with the forepaws, and scratching with any limb. Grooming was usually performed in a sitting position.
Maternal behavior	Nursing	Incorporates both active and passive nursing; At least one pups is attached to a dam's teat and actively nursing.
	Nest building	Any behavior where an adult has some contact with nesting material or bedding and appears to be altering the morphology of the nest site. Can include pushing, fraying, and pulling in materials around pups.
	Licking or grooming pups	All grooming behavior as listed above but is directed at the pups
	Retrieving pups	A pup that is no longer in the nest site is carried back to the nest site by an adult
Aggressive behavior	Mediated	Mounting; Crouch; Elevated crouch; Submissive upright
	Escalated	Attack; Fighting
Unknown	Unknown	An animal inside or outside of the nest but unsure of the behavior being displayed or the view of the animal is blocked.
	Unknown-in-nest	An animal inside of the nest but unsure of the behavior being occurring inside of the nest. This is different from nest building in that the nest does not appear to be growing. This is also different from inactive in nest (IN) in that movement is seen within the nest.

This ethogram was used in the study for instantaneous scan sampling every 30 minutes of a 24 hour period of video.

**Table 4:** Ethogram for 1/0 sampling.

Press posture		Male or female is seen to wedge the ventral surface of its body (at least half) in the corner of the cage. Eyes may be open or closed but the animal must remain in this position for at least 5 seconds.
Stereotypy*	Bar mouthing	Mice hold a cage bar in their diastema, and either a) make a series of sham-biting movements down the bar; or b) wipe their open mouth along the bar; or c) wipe their open mouth along the bar whilst rotating or flicking their head backwards and forwards.
	Jump	Jumping up towards the cage lid. Intense repetitive bouts of jumping are considered a stereotypy (more than 10 jumps per minute).
	Route tracing	The animal traces out an identical repeated route around the cage.
	Looping	The animal turns back-flips either in open space or against the wall of the cage.
	Twirling	The animal hangs from the cage-roof by its forepaws, and moves in rapid tight-circles, the hind-limbs may or may not be used in individual animals.
Play	Popcorn	Pup appears to leap vertically with all 4 feet and may be combined with rapid locomotion to or from siblings

*If there are brief interruptions (5 seconds or less) in the stereotypy by grooming, sniffing, or pausing, that can still be considered a bout of stereotypy.

Video was observed for 2 minutes every 30 minutes over 24 hours and the presence/absence of these behaviors was scored.

Location 2 was used to model a barrier room setting and focused primarily on reproduction. As with the behavior portion of the study, the data collected included: litter size at birth, litter size at weaning, and weaning weight, and whether females were pregnant at the end of the study. From those values, we calculated the interlitter interval between the first and second litter and the number of pups weaned/female/week (PI). Animals were assigned to treatment groups using the random integer set generator function found at random.org. A total of 240 mouse cages were set up with 560 mice, divided evenly between B6 and CD1. Cages were allocated to breeding treatment as follows: 80 cages (40 CD1 and 40 B6) as trio matings with 1 male and 2 females; 80 cages (40 CD1 and 40 B6) were pair-mated with 1 male and 1 female; and finally, 80 cages (40 CD1 and 40 B6) in which the male was removed 24–48 h after a litter was born, to model a breeding situation in which the female rears the pups by herself. For solo females, the male was reintroduced after the pups were weaned only if the female had not become pregnant at the post-partum estrus. Simple behavioral data were collected on the day of weekly cage change. The data were limited to noting hair loss (yes/no) or fight wounds (yes/no) when animals were handled during cage change and then observing animals for stereotypies during a 5 second scan of each cage 30 minutes prior to the end of the day in 1/0 sampling. If any adult mouse in a cage exhibited a stereotypy during the observation period, the cage was scored as a 1. Mice were considered stereotypic if they were seen displaying the behavior three times or more within the scan time period. Nest scores were also collected weekly based on a 1–5 scale [40].

All statistical analyses were conducted in JMP v 9 (SAS, Cary, NC). The likelihood of pregnancy at the end of the experiment was analyzed as a Generalized Linear Model (GLIM). The rest of the analyses were run as an ANOVA using a General Linear Model (GLM). The assumptions of GLM (normality of error, homogeneity of variance, and linearity) were confirmed *post-hoc* [41]. Significant effects were then analyzed using *post-hoc* Tukey tests or Bonferroni corrected planned contrasts using custom contrasts in JMP. Cage treatment in all analyses was treated as a categorical variable due to the fact that many aspects of these commercially available cages differed (for example, different dimensions, shape, and depth of feed hoppers) and this may have influenced results along with total floor space.

For reproductive results, data from both Location 1 and Location 2 were included in the analysis, incorporating non-reproductive cages, but blocked by location in the model (number of cages per combination listed in Part A of S1 Table). Non-reproductive cages were not included in behavioral data because pups must be present in order to observe interactions between adults and offspring. A full factorial model between strain, breeding treatment, cage treatment, and location was initially tested. Due to non-orthogonality of the dataset, insignificant interactions were removed from the model [41]. Average weaning weight per litter utilized a similar model as above but was blocked by cage, and nested within strain, breeding treatment, and cage treatment. Only data from reproductively successful cages were included in the analysis of average weaning weight. The model also included litter number and number of pups born alive as covariates. Square root transformed pup mortality was calculated per cage as 1-(number of pups weaned/number of mice born, either dead or alive). Interlitter interval was calculated for each individual female by calculating the number of days for cages with 2 or more litters. The interlitter interval was averaged per cage in trio matings. Only 268 cages from both Location 1 and Location 2 produced 2 or more litters over the 12 weeks. Therefore, the final n per combination is provided in Part B of S1 Table. All values are given as least squares means and standard error.

Adult population time budgets were only calculated for the Location 1 mice that produced litters. The total number of times each category of behavior or location within the cage (i.e. under feeder or other) for each day was divided by the total number of observations for that group. Following this calculation, data from the ethogram category unknown behaviors were excluded from the analysis. Thus the behavioral time budget does not total 100% and the independent variables are not co-linear. In essence, change in one behavioral category will not directly influence the level of another behavioral category. A factorial model to the 3^rd^ order interactions between strain, breeding treatment, cage treatment, PND of pups, and behavior was initially tested. For datasets that were not orthogonal, non-significant high order interactions were removed from the model [41]. Data were blocked by cage and nested within strain, breeding treatment, and cage treatment. Behavioral budget, press posture, and play behavior were angularly transformed for normality. Number of pups in the cage was tested as a covariate for both 1/0 scored behaviors but was not significant for either analysis and was removed from the model. Nest score was tested similarly by cage being nested within strain, cage treatment, and breeding treatment with all 2^nd^ order interactions as well as the strain, cage treatment, and breeding treatment interaction. Litter in the nest as well as litter in the nest by breeding treatment was tested. The model for stereotypy included main effects for strain, breeding treatment, cage treatment, and rack as a block. Hair loss was run as a binary logistic regression with Firth bias adjustment also testing main effects and the block of rack.

## Results

### Reproduction

From the total of 320 cages assessed for reproductive parameters between the two locations, four B6NCrl females were found dead within the first few weeks of the experiment in Location 1of unknown causes and one was euthanized due to dystocia. These five cages along with six cages from Location 2 excluded for missing data were not included in the final analysis. Six cages (1 from Location 1 and 5 from Location 2; all B6NCrl cages) produced no litters and 268 cages had 2 or more litters (120 B6NCrl cages and 148 CD1 cages).

The total number of pups born per cage was affected by a 2nd order interaction between the strain of mouse and breeding treatment (GLM: F_2, 297_ = 27.3; P < 0.001). In CD1 mice, all three breeding treatments differed significantly from each other in pups born (trio: 62.8 pups/cage ± 1.5; pair: 38.5 pups/cage ± 1.3; single: 32.9 pups/cage ± 1.4; P < 0.05). In B6NCrl cages significantly more pups were born in trio cages than pairs and singly housed mice (B6NCrl trio: 24.1 pups/cage ± 1.4; B6NCrl pair: 14.8 pups/cage ± 1.3; B6NCrl single: 14.8 pups/cage ± 1.3; P < 0.05). In Location 1, more pups were born per cage than in Location 2 (GLM: F_1, 297_ = 21.0; P < 0.001; Location 1: 34.0 pups/cage ± 1.1 vs Location 2: 28.2 pups/cage ± 0.6).

The number of pups weaned was similarly affected by strain and breeding treatment (GLM: F_2, 297_ = 28.1; P < 0.001). All breeding treatments in CD1 were significantly different from one another (CD1 trio: 60.6 pups/cage ± 1.5; CD1 pair: 37.9 pups/cage ± 1.3; CD1 single: 31.9 pups/cage ± 1.4). In B6NCrl cages, trio mated cages weaned significantly more pups than other breeding treatments (B6NCrl trio: 21.5 pups/cage ± 1.4; B6NCrl pair: 13.0 pups/cage ± 1.3; B6NCrl single: 12.5 pups/cage ± 1.4). Again, a greater number of pups per cage were weaned in location 1 than in location 2 (GLM: F_1, 297_ = 12.96; P < 0.001; Location 1: 31.8 pups/cage ± 1.1 vs Location 2: 27.3 pups/cage ± 0.6). CD1 pups were significantly heavier at weaning than B6NCrl mice (GLM: F_1, 531_ = 600.7; P < 0.001; CD1: 12.7 g ± 0.1 vs B6NCrl: 8.2 g ± 0.1). Unsurprisingly, a negative correlation between number of pups in the litter and the average pup weaning weight was significant (GLM: F_1, 531_ = 166.0; P < 0.001). Thus, pups from larger litters weighed less at weaning than those from smaller litters. The covariate of litter number also had a negative effect on pup weaning weight (GLM: F_1, 531_ = 98.1; P < 0.001).

PI was not affected by any of the cage sizes (GLM: F_3, 294_ = 0.30; P = 0.83). Breeding treatment only affected the PI of CD1 pair housed mice, which was significantly higher than both the trio and singly housed mice (CD1 trio: 2.6 pups weaned/female/week ± 0.09; CD1 pair: 3.2 pups weaned/female/week ± 0.08; CD1 single: 2.7 pups weaned/female/week ± 0.09; P < 0.05). PI was not affected by breeding treatment in B6NCrl mice (P > 0.05). Interlitter interval was affected by strain, where B6NCrl mice had a slightly longer interval than CD1 mice (CD1: 24.0 ± 0.92 days vs B6NCrl: 33.3 ± 0.99 days; GLM: F_1, 254_ = 55.4; P < 0.001). Mice at Location 1 also had a significantly shorter interlitter interval than at Location 2 (Location 1: 26.6 ± 1.2 days vs Location 2: 30.7 ± 0.73 days; GLM: F_1, 254_ = 8.4; P = 0.004). Pup mortality was higher in B6NCrl mice than CD1 (GLM: F_1, 254_ = 31.8; P < 0.001; CD1: 12.1% ± 1.8% vs B6NCrl: 25.4% ± 1.8%) and was also significantly higher in trio vs singly bred mice (GLM: F_2, 254_ = 4.17; P = 0.016; trio: 23.1% ± 2.2%; pair: 18.6% ± 2.1%; single: 14.6% ± 2.6%). A higher percentage of pup mortality was also found in Location 1 (GLM: F_1, 254_ = 14.8; P < 0.001; Location 1: 24.1% ± 2.4% vs Location 2: 13.5% ± 1.4%). Finally, CD1 mice were more likely to be pregnant at the end of the study than were B6NCrl mice (χ2 = 38.4; P < 0.001). Sixty-three B6NCrl and 113 CD1 females were found pregnant at the termination of the study. An interaction between breeding treatment and cage size was found (χ2 = 23.3; P < 0.001). In trio bred mice, fewer mice in the A N10 cage were pregnant than in the A RC1 (contrast: α/6; P = 0.005). Pair housed mice had the highest likelihood of being pregnant in the A N10 cage, which was significantly higher than the A RC1 (contrast: α/6; P = 0.001) and the T 1291 (contrast: α/6; P = 0.006) cages. Mice at Location 2 were also more likely to be pregnant at the end of the study than at Location 1 (χ2 = 6.11; P = 0.01).

### Behavior

**Fig 3:**
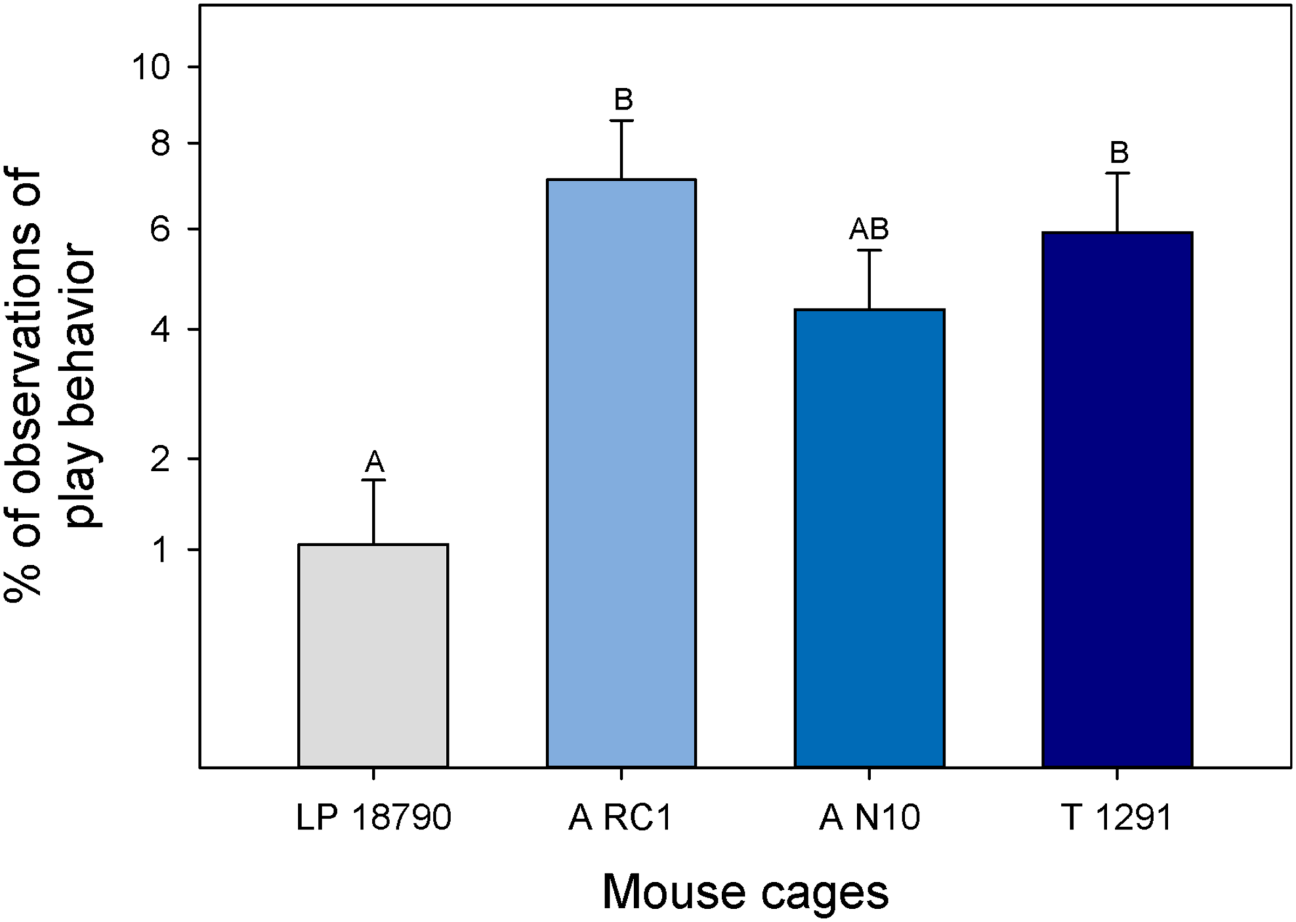
Proportion of play behavior observed in the different cage sizes. Least square means and standard errors are plotted against an angularly transformed y-axis. *Post hoc* Tukey tests were considered significant if P < 0.05. Different letters indicate statistical differences (P < 0.05).

**Fig 4:**
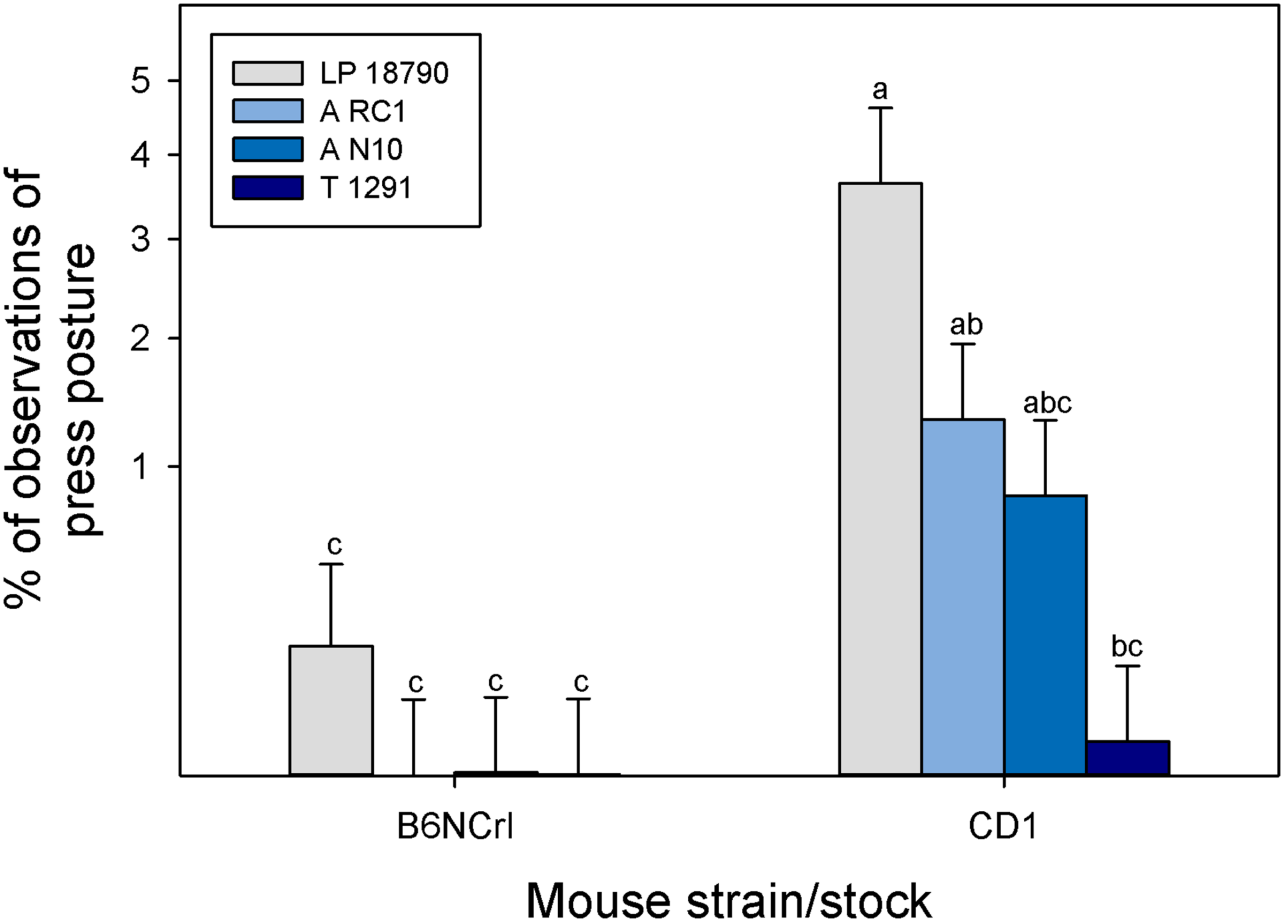
Proportion of press posture observed in different sized cages for the two types of mice tested. *Post hoc* Tukey tests were considered significant if P < 0.05. Least square means and standard errors are plotted against an angularly transformed y-axis. Different letters indicate statistical differences (P < 0.05).

**Fig 5:**
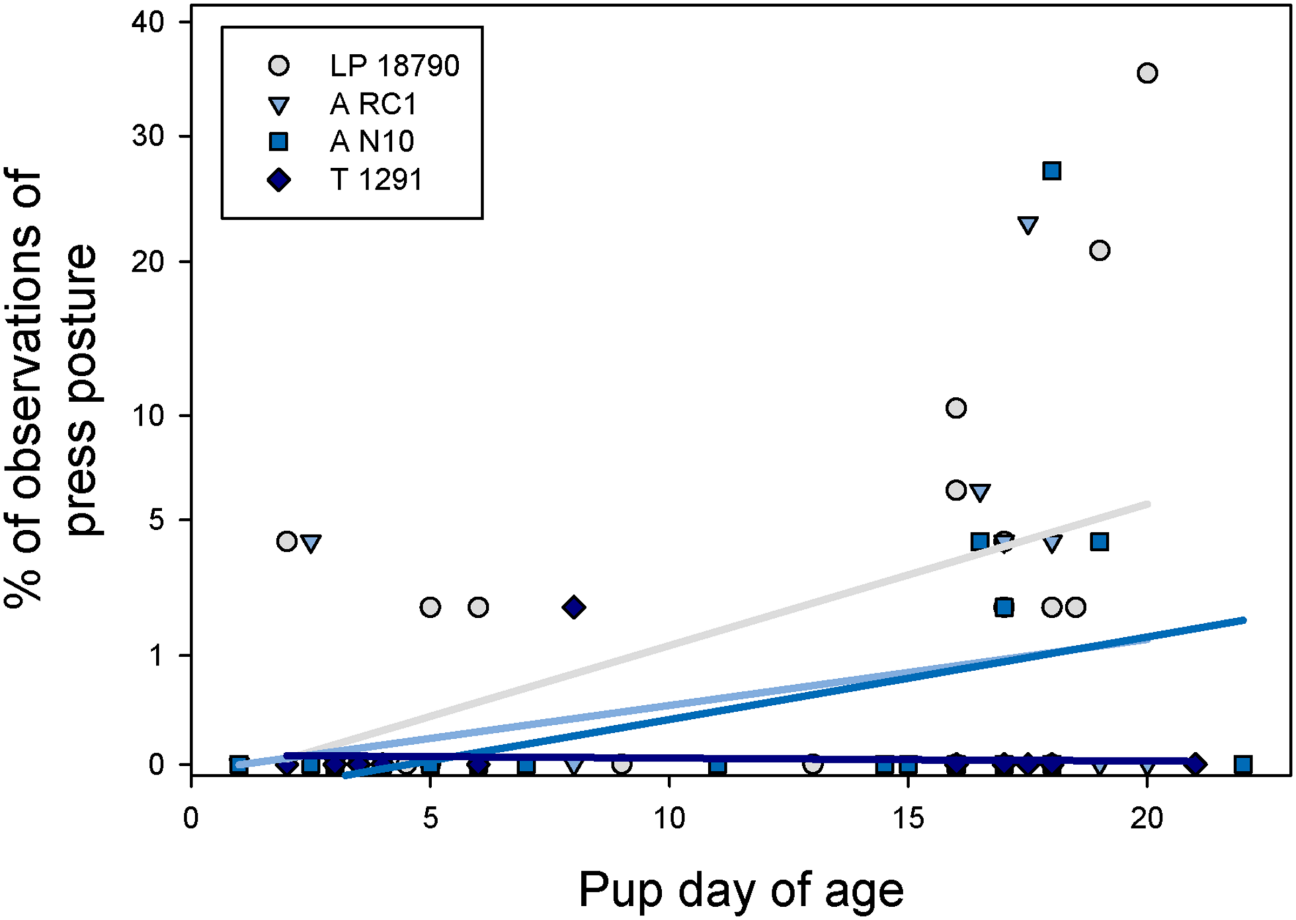
Proportion of press posture observed at different pup ages in the four cage sizes. Data points are plotted with the least squares line.

No significant results were found based on any of the treatments for the general behavioral budget and no stereotypic behaviors were observed in the Location 1 animals. However, cage treatment did affect play behavior in pups (GLM; F_3, 23_ = 6.61; P = 0.002; Fig 3). Pups housed in the LP 18790 cage (the smallest cage) were observed playing less than pups in the A RC1 (second largest) and T 1291 (largest) cages (P < 0.05). Breeding treatment also affected the amount of play behavior but was dependent on strain (GLM; F_2, 23_ = 6.41; P = 0.006). More play behavior was observed in pups from CD1 mice bred as trios (28.3% ± 3.3%) compared to single female cages (11.9% ± 3.3%; P < 0.05). Cage treatment affected press posture but only in CD1 mice (GLM; F_3, 42_ = 2.85; P = 0.04; Fig 4). CD1 mice in the smallest cage (LP 18790) spent more time in press posture than in the largest cage (T 1291; P < 0.05). Breeding treatment also affected press posture but again only in CD1 mice (GLM; F_2, 42_ = 5.63; P = 0.006). Press posture was observed more frequently in trio cages (trio: 19.0% ± 2.1%; pair: 9.0% ± 2.1%; single: 2.7% ± 2.1%; P < 0.05). As pups got older, there was a significant increase in observed press posture in the two smallest cages (GLM; F_3, 42_ = 4.18; P = 0.01; Fig 5). The slope of the predicted line was only significant for the LP 18790 (Custom test: α/4: F_1, 42_ = 21.2; P < 0.001) and A N10 cages (Custom test: α/4: F_1, 42_ = 7.83; P = 0.008). Press posture was similarly increased when pups were older in CD1 mice (GLM; F_1, 42_ = 9.95; P = 0.003; Custom test: α/2: F_1, 42_ = 34.6; P < 0.001) but age did not alter the frequency of this behavior in B6NCrl mice (Custom test: α/2: F_1, 42_ = 1.56; P = 0.21).

**Fig 6:**
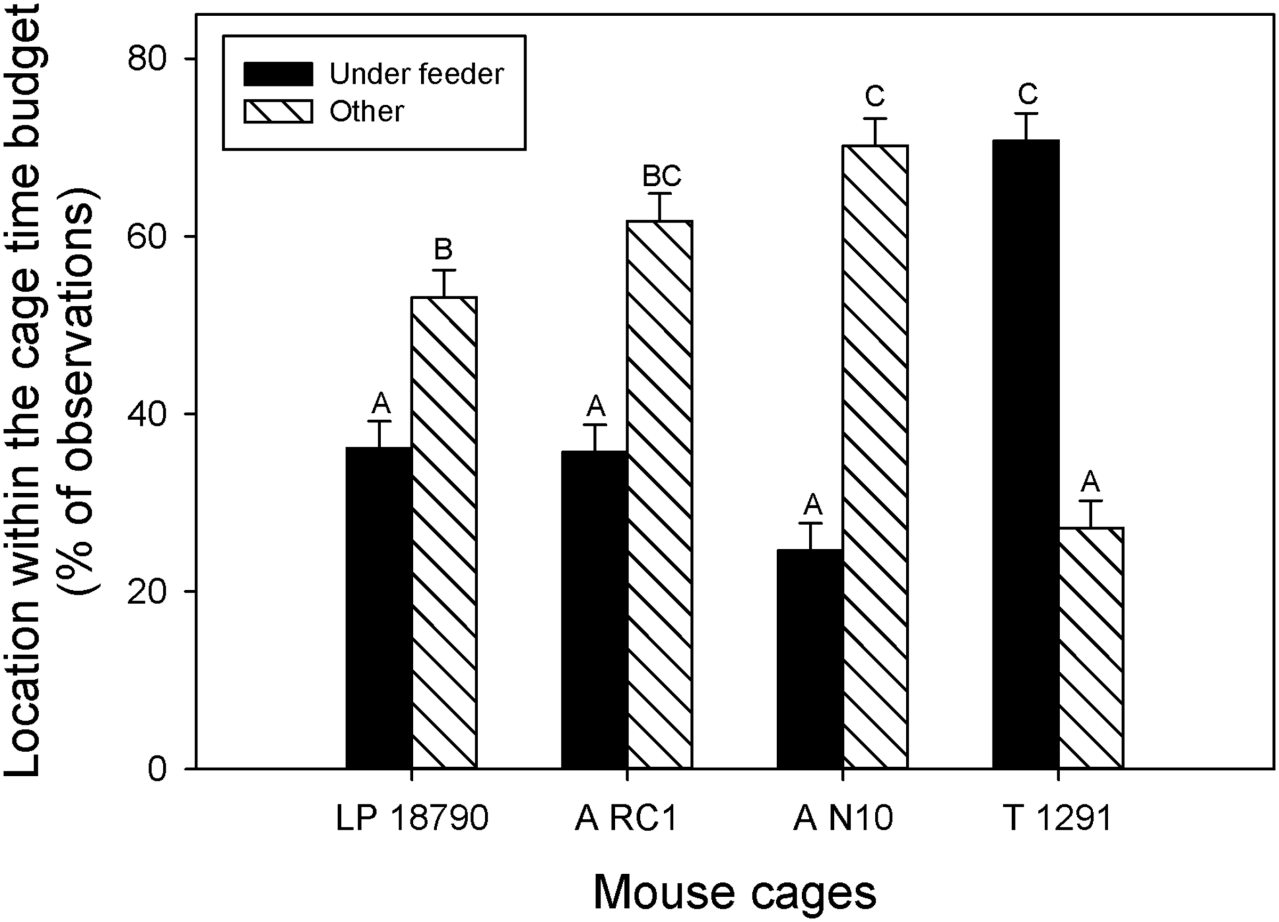
Location preference within the cage. Least square means and standard errors are plotted against the y-axis. *Post hoc* Tukey tests were considered significant if P < 0.05. Different letters indicate *post hoc* statistical differences.

The proportion of time the mice spent in different areas of the cage varied with breeding treatment (GLM; F_2, 110_ = 5.9; P = 0.004), cage treatment (GLM; F_3, 110_ = 78.4; P < 0.001), and the age of pups (GLM; F_1, 110_ = 6.4; P = 0.013). Pair housed mice spent significantly more time in areas of the cage other than under the feeder (P < 0.05). Mice housed in the T 1291 cage were observed under the feeder more often than in the other areas of the cage, but the opposite was found for the other cages (Fig 6). Lastly, mice during PND 0–8 were more likely to be found in other areas of the cage (under the feeder: 40.4% ± 2.1% vs other: 57.2% ± 2.1%; P < 0.05) but no differences were found at PND 14–21 (P > 0.05).

**Fig 7:**
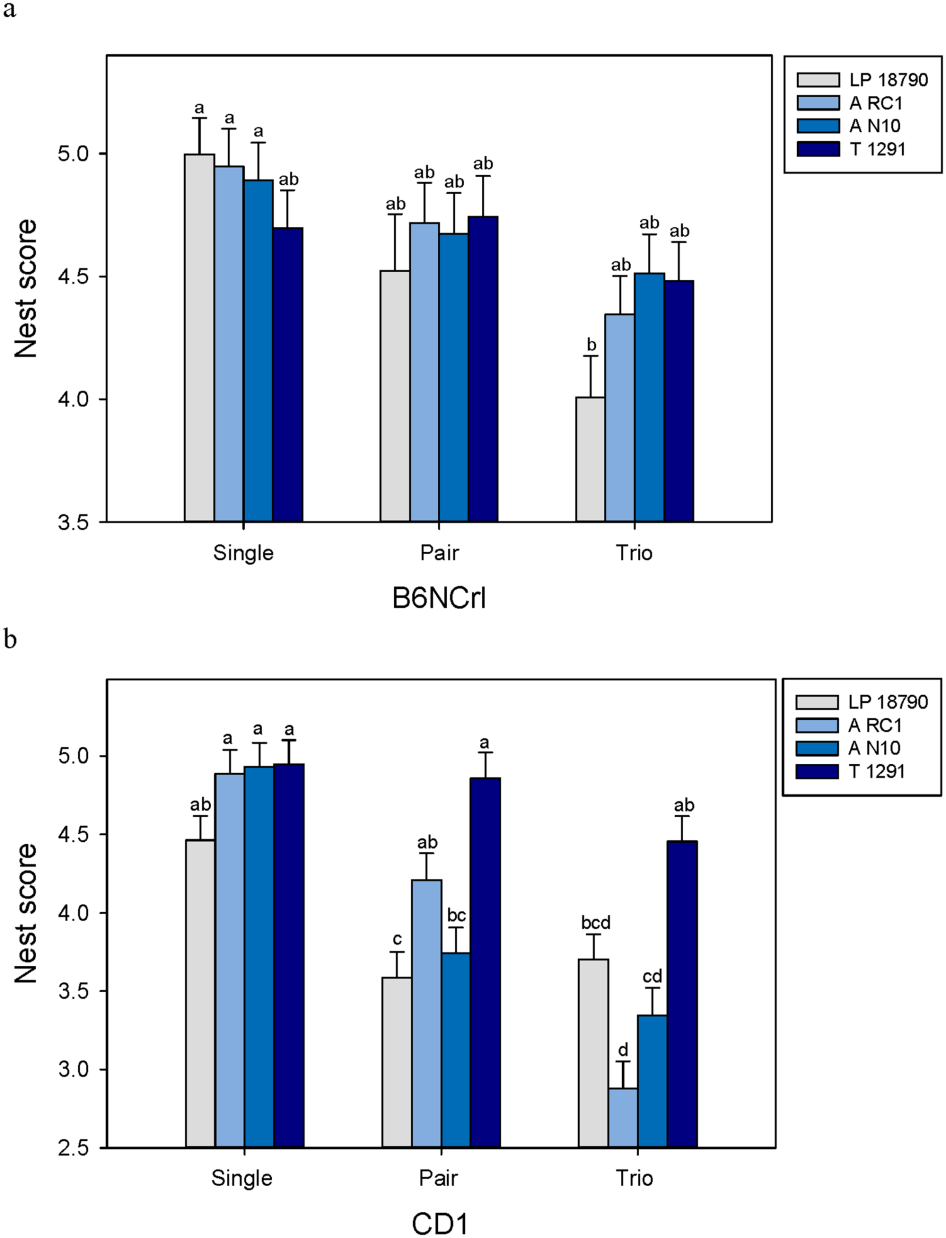
Differences in nest score observed in different cages for a) B6NCrl mice and b) CD1 mice. Least square means and standard errors are plotted against the y-axis. Different letters indicate statistical differences based on Tukey pairwise comparisons.

Weekly nest scores were significantly altered by a 3^rd^ order interaction between mouse strain, cage treatment, and breeding treatment (GLM; F_6, 408_ = 3.62; P = 0.002). Very few differences in nest score were found for B6NCrl mice (Fig 7a) except in the smallest cage (LP 18790) between mice bred singly and as trios (P < 0.05). Many more differences were found between treatment cages for pair and trio breeding in CD1 mice (Fig 7b). The presence of a litter in the nest as well as breeding treatment also altered overall nest quality (GLM; F_2, 408_ = 3.61; P = 0.03). Test slices, however, did not reveal a truly significant difference between the breeding treatments.

Live behavioral observations for stereotypy were made in Location 2 of the experiment. CD1 mice were found to display more stereotypic behavior during observations than B6NCrl mice (CD1 12.7% of weekly observations ± 1.7%; B6NCrl 2.0% of weekly observations ± 1.7%; GLM; F_1, 157_ = 10.8; P = 0.001). Additionally, mice in the largest cage (T 1291) displayed more stereotypies than the second smallest cage (A RC1; GLM; F_3, 157_ = 3.4; P = 0.019). B6NCrl mice on the other hand, had more observed hair loss than CD1 mice (χ^2^ = 3.8; P = 0.048). Breeding treatment also significantly affected hair loss (χ^2^ = 14.9; P < 0.001). Singly housed females had a lower occurrence of hair loss than pair (Custom test; α/3 χ^2^ = 14.7; P < 0.001) or trio bred cages (Custom test; α/3 χ^2^ = 6.5; P = 0.01). Only 2 mice were found wounded over the whole experiment and data on wounding were therefore not analyzed.

## Discussion

As expected, differences in fecundity associated with mouse background were the primary differences found in the study. Outbred CD1 mice are significantly more productive than inbred B6NCrl mice, with a greater number of overall pups produced, a shorter interlitter interval, and a higher production index. One interesting finding is that the calculated production index per female was greater in pair-bred CD1, although the overall production per cage was higher per cage in trio-bred CD1. This indicates that in this experiment, production per CD1 female was maximized with pair breeding, but production per CD1 cage was maximized with trio breeding. No such difference was seen in the production index of B6NCrl mice when examined by breeding condition and in fact, significantly more pups were born and weaned from B6NCrl trio cages when compared to pair or single cages. Pup mortality was found to be significantly affected by strain, breeding treatment, and location. Higher pup mortality was seen in the B6NCrl mice, trio mated animals, and Location 1. Regardless of strain, some pups are likely to be stillborn [42], B6NCrl have more perinatal mortality than other strains [42], and mating animals in trios may result in mortality of pups due to trampling of young pups by older animals or younger pups not being able to effectively compete for teat space. CD1 dams had more and heavier pups than B6NCrl mice, but in both strains, litter size was negatively correlated with weaning weight. This is a well-known phenomenon in litter-bearing animals [43]. The only interaction between reproductive measures and cage size was seen in trio and pair bred animals, but were contradictory. In pair bred mice, more animals in the A N10 cage (3^rd^ of 4 on a scale of smallest to largest) were pregnant at the end of study, while in trio mice, animals in the A RC1 (2^nd^ of 4 on a scale of smallest to largest) were more likely to be pregnant. These findings are not easily explained by space available and may be related to factors we could not change due to the nature of the study such as the shape and location of the feeder.

There were several significant differences found between Locations 1 and 2, including whether or not a female was pregnant at the end of the study, number of pups born, pup mortality, and number of pups weaned. The location effect on pregnancy, pup number, and mortality was unexpected and not easily explained, but the follow on from an increase in pups born, an increase in pups weaned, is logical. Gross environmental measures were similar between locations, including the presence of rats in the room, the light cycle, and the temperature. There may have been other environmental influences difficult for humans to detect such as drafts, magnetic fields, or ultrasonic noise and these may have differed between the locations. One known difference in Location 2 is that a caretaker was present in the room nearly full-time to manage the care of the large number of cages. In Location 1, the presence of caretakers was intermittent and brief, other than on cage changing days. Even then, the cage changes could be accomplished much more quickly with the smaller number of cages in Location 1. Perhaps the continued presence of caretakers in an animal housing situation, even a stable, familiar caretaker, is inhibiting to reproduction in mice. The increased mortality at Location 1 might be due to environmental or caretaker influences, or due to the fact that for unknown reasons, more pups were born than the lactational capacity of the mother could support.

In the focused behavioral evaluation, which was conducted entirely from video recordings taken in Location 1, there were no significant differences in the overall time budget and no stereotypic behaviors were observed. Lower levels of play behavior were also seen in smaller cages in the focal animals. This is likely related to the crowded conditions found in the smallest cage as the animals reached the age when play behavior typically occurs and did not possess the space needed to display the behavior [44].

In Location 2, adult CD1 mice showed higher levels of stereotypic behavior in the largest cage. Environmental complexity is known to reduce the occurrence of stereotypy development but cannot completely eliminate stereotypic behavior [45]. Unfortunately the function or exact conditions that contribute to stereotypy development are unknown. Stereotypies, such as bar chewing jumping, or twirling, were observed in a rapid scan sample due to the large number of cages on study. It is possible that the rate may be inflated due to the inability to observe cages for longer periods of time and therefore misclassification of observed behavior based on the ethogram provided. Since many stereotypic behaviors in mice are likely rooted in escape or exploration behaviors [46–48], perhaps the lack of space in smaller cage induces learned helplessness (or behavioral despair) instead of stereotypy development. Learned helplessness, a form of depression or apathy seen in humans and animals, derives from the expectation that there are no relationships between available responses and consequences [49] In the case of this experiment, conditions in smaller cages may have impaired the learning of escape strategies because this environment did not allow for this response. Larger cages may also give more opportunity for locomotor stereotypies while in smaller cages the stereotypies may be less obvious. Humans in high-density residential settings exhibit more symptoms of learned helplessness than those from small residential groups [50]. Nevison et al. [51] similarly found an unexpected decrease in stereotypic behavior in mice from barren cages rather than from enriched cages. It was similarly hypothesized that the barren conditions may have induced behavioral despair, potentially affecting the development of stereotypic behavior [49]. Another abnormality noted was hair loss in adult animals. B6NCrl mice had higher levels of observed hair loss when compared to CD1 mice, and the hair loss was least in individually-housed females, but did not differ between pair and trio breeding. The hair loss was likely due to barbering, or hair-pulling behavior, a behavior difficult to observe while occurring, but the consequences of which are readily apparent. These findings are expected in that B6 background mice are known to perform hair-pulling behavior and that self-barbering is less common than barbering others [52,53].

In this study, we did observe one novel behavior not previously described in mouse ethograms which we termed press posture, after a similarly-described behavior in rats [54,55]. (Fig 2). In the two published descriptions of this behavior in rats, one was observational, noting that the behavior occurred [54], while the second study noted that the behavior occurred even though the rats were allowed to escape their young through cage dividers [55]. Cramer et al. [55] hypothesized that the behavior was due to females initiating weaning by preventing the pups from nursing or attempting to increase heat loss by pressing metabolically active mammary tissue against the cage wall. This behavior was not originally part of the ethogram used to evaluate the mice, but when data were being collected it was seen repeatedly, so the ethogram was amended to include this behavior and previously viewed videos were re-evaluated. Both males and females were observed exhibiting press posture and this behavior appeared to be an attempt to remove themselves from direct interaction with the pups. Animals were seen to fall asleep in this position, so this position may be a way for the adults to obtain relatively undisturbed rest in the smaller cages. Inability or disinclination to sleep in normal postures may be an indicator of stress or crowding in these smaller cages. While we cannot directly link press posture or its frequency to stress, the behavior appears to increase with decreased floor space, potentially indicating some sort of space oriented stress. Press posture was also exhibited by the B6NCrl mice, but there was no significant difference in the occurrence of this behavior based on cage size. In the CD1 mice, the onset of this behavior was related to both the age of the pups in the cage and the size of the cage. Press posture was rarely seen in the first week after the birth of a litter, and if it was seen, it was more likely to occur in the smallest cage (Fig 4). As pups grew, especially in the smaller cages, the frequency of the behavior increased (Fig 5).

When the overall behavioral budget was examined to determine where the animals spent the most time in the cage, their physical location within the cages varied, with mice housed in the largest cage spending more time under the feeder than mice in the other 3 cage sizes. The largest cage is also the tallest cage and this locational preference may be related to mouse anxiety in open spaces. In the largest cage, this was the most “protected” area within the cage. It is important to note that this study used only commercially available cages, not cages constructed specially for the experiment. All the cages were purchased directly from the manufacturer and were not altered with the exception of removing filter material from the lid of the LP 18790 cage. All cages in this study differed in height, had different feeder capacities and configurations, and had different water bottle orientations (some faced a wall of the cage, others faced the interior). There may be interactions between the mice and the cage other than simply available cage space.

The presence of nesting material in every cage may explain why this study did not show improved breeding performance in smaller cages as other investigators have [7]. Mice in the laboratory are cold stressed [56] and this has been shown to alter reproduction [57]. In larger cages, total cage body mass is spread out, reducing the animals’ ability to conserve heat. The nesting material provided would help eliminate thermal stress in larger cages while animals in smaller cages that would not need the insulation from nesting material would not use it, thus normalizing the effects of thermal stress (Fig 7). To further support this theory, we found higher nest scores in larger cages, indicating that animals built more thermally effective nests.

The present study shows that laboratory mice can successfully breed in a variety of cage sizes. However, there seem to be behavioral indicators, such as an increased frequency of press posture, that some of the cages we tested crowd breeding mice. Crowding should be distinguished from density [58] and the simplest way to consider it is that density is defined solely by space available, while crowding is a motivational state which occurs through the interaction of spatial and social factors, with motivation directed toward ending the perceived spatial restriction. By increasing the frequency of press posture, adult CD1 mice in the smallest cages would seem to be exhibiting a behavior associated with crowding. In other intensive production situations, such as mink breeding, allowing females to escape their litters by utilizing a platform or a upper level of a cage increases production and reduces pup mortality [59,60]. Breeding rats will use elevated platforms and multilevel cages to modulate proximity to pups [61] and it is not unreasonable to think that mice might also benefit from the ability to choose whether to interact with pups.

In addition, the bedding in the smallest cage housing trios with litters, especially CD1 mice, became heavily soiled within hours of changing. This soiling required more frequent cage changes in order to provide the animals with an environment that is clean and esthetically pleasing to humans [31,62,63]. Although mice do not necessarily avoid feces [64], the *Guide* also suggests that animals have areas to rest which are free of fecal matter. The smaller cages also may require more daily management to be sure animals have access to appropriate amounts of food and water, since the food hopper and water bottle are both proportional to the size of the cage.

In this study, we were interested in observing and quantifying the behavior of two commonly used “breeds” of mice set up in typical reproductive groups, and measuring their reproductive output as a way to examine stress. We chose these methods based on their practicality and relevance to the question at hand (does cage space affect mouse breeding?) as well as their potential to influence how animals are managed. Methods that require complicated analyses or sophisticated equipment will be limited in their applicability and reproducibility [65]. Biochemical methods, such as serum or fecal corticosterone levels, are subject to normal fluctuations based on circadian rhythms [66,67], and the literature on corticosterone levels in unstressed pregnant mice are from studies in animals that are singly housed, a source of stress for mice [5,68]. Examination of physiological measures may not have differentiated between stress induced by crowding and the normal stresses of pregnancy, parturition, and lactation. We also chose to avoid excess handling since mice will acclimate to procedures [69,70], and this could also change behavioral measures throughout the study [32]. The authors do acknowledge that biological functioning as indicated by successful reproduction is not considered by all to be a definitive measure of welfare in a highly fecund prey species that that has been domesticated and further selected for this trait under a variety of conditions [65]. Measuring reproductive output was deemed appropriate, biologically relevant, reproducible for this scientific question, and if reproduction had been negatively affected, would have indicated poor welfare.

In this study, we did not query the mice as to their preference in cage size. Breeding makes asking how much space is required for a group of mice difficult, as offspring are fully dependent on parents until they are mobile, parental care extends until weaning, and preferences of parents may differ from those of offspring. Near weaning, offspring occupy more physical space within the cage, which may alter the interactions of the breeding group. Attempting to ascertain the preferences of breeding mice for cage space has not been done in a systematic manner and this would be difficult to accomplish in commercially available caging. When examining the preferences of animals, consideration should also be given to the intended purpose of the animals; they may prefer conditions that make their captivity (and freedom from disease, hunger, thirst, and predation) impractical, but not show a strong preference when asked to choose between conditions that can be made reasonably available in the laboratory [71]. Preference testing, although a valuable way of asking questions of animals, should always be placed in context, as animals will choose things they prefer in the short term, but that may be detrimental over the long term, just as humans will [72].

A few extra square centimeters of space is unlikely to be crucial to reproductive success or improved welfare for most strains or stocks of mice and may be detrimental under normal laboratory temperatures if nesting material is not made available. Laboratory housing makes it impossible to provide mice with the square meters of territory that a breeding group might patrol in the wild, but that should not be taken to mean that less space than is customary is necessarily preferable, or that animals should routinely be bred in the smallest cage we used. Animals should also be able to express normal postures and have freedom of movement; both conditions were difficult to achieve in the smallest cage when three adults and two litters were present. Our findings were that only in the smallest cage housing the largest mice were there any possible behavioral indications of stress, and that for cages that were either 1/3 smaller or 2.5 times larger than the current *Guide* recommended floor space for breeder groups, there were no biologically significant differences in reproductive performance. This study was not able to identify an amount of cage space in which mice would not reproduce (a positive control for the experiment). In many facilities, the positive control for evaluating mouse space needs is “this appears intolerable to human observers”, which is a highly subjective measure. The biggest difference found was between animals of the two different genetic backgrounds that we studied. The large outbred CD1 mice differed in one behavioral indicator that the authors believe is associated with crowding in the smallest cage while the smaller inbred B6NCrl did not. This should not be taken to mean that it is necessary to study the cage space needs of breeding mice of every strain in every facility, because the spread in adult size and pup number between CD1 and B6NCrl covers most inbred and outbred mice in use today, and addressing every single mouse strain created would be prohibitively expensive. Animals that are more fecund than the CD1 are rare and animals that are less fecund than the B6NCrl would be likely to be even less affected by less space, since they typically have smaller litter sizes and longer interlitter intervals. Cage space properly belongs as a performance standard, not an engineering standard. Rather than simply increasing space and assuming welfare will also increase, increasing the biologically relevant cage complexity may be of more value to mice and is the challenge faced by laboratory animal scientists and caging manufacturers.

## Supporting Information

S1 Table A) The number of cages used in cage level reproductive data analysis. The table is split between the 2 locations to better illustrate the number of cages per treatment combination. B) The number of cages used in the analysis of interlitter interval. Data was only included from cages which had more than 1 litter and only the interval between the first and second litter was analyzed. The table is split between the 2 locations to better illustrate the number of cages per treatment combination in both locations.
(DOCX)

S1 Dataset Raw reproductive data at both the cage level and separated out for each litter in the two locations. Raw behavioral data for 1–0 scoring (Play and Press Posture), General behavior, and Location within the cage are provided. Raw weekly nest scores per cage are also provided.
(XLSX)
