# Supplementary material for: The Effect of Cage Space on Behavior and Reproduction in Crl:CD1(Icr) and C57BL/6NCrl Laboratory Mice
Source: PLoS One. 2015 May 28;10(5):e0127875. doi: 10.1371/journal.pone.0127875 (PMC4447268; doi:10.1371/journal.pone.0127875)
Supplement: S1 Table — A) The number of cages used in cage level reproductive data analysis. The table is split between the 2 locations to better illustrate the number of cages per treatment combination. B) The number of cages used in the analysis of interlitter interval. Data was only included from cages which had more than 1 litter and only the interval between the first and second litter was analyzed. The table is split between the 2 locations to better illustrate the number of cages per treatment combination in both locations. (DOCX) [file pone.0127875.s001.docx]

**Table S1**

A)

Cage level reproductive data collected and analyzed from the number of cages listed below.

|  | Location 1 | | Location 2 | |
| --- | --- | --- | --- | --- |
|  | B6NCrl | CD1 | B6NCrl | CD1 |
| LP 18790 | 7 | 11 | 30 | 29 |
| A RC1 | 9 | 10 | 28 | 30 |
| A N10 | 12 | 8 | 31 | 27 |
| T 1290 | 10 | 8 | 29 | 28 |

B)

Interlitter interval data collected and analyzed from the number of cages listed below.

|  | Location 1 | | Location 2 | |
| --- | --- | --- | --- | --- |
|  | B6NCrl | CD1 | B6NCrl | CD1 |
| LP 18790 | 7 | 10 | 24 | 29 |
| A RC1 | 7 | 10 | 19 | 29 |
| A N10 | 9 | 8 | 18 | 25 |
| T 1290 | 10 | 8 | 26 | 28 |
